# Optically controlled magnetic-field etching on the nano-scale

**DOI:** 10.1038/lsa.2016.54

**Published:** 2016-03-25

**Authors:** Takashi Yatsui, Toshiki Tsuboi, Maiku Yamaguchi, Katsuyuki Nobusada, Satoshi Tojo, Fabrice Stehlin, Olivier Soppera, Daniel Bloch

**Affiliations:** 1 School of Engineering, University of Tokyo, Bunkyo-ku, Tokyo, 113-8656 Japan; 2 Department of Theoretical and Computational Molecular Science, Institute for Molecular Science, Myodaiji, Okazaki, 444-8585 Japan; 3 Faculty of Science and Engineering, Chuo University, Bunkyo-ku, Tokyo, 112-8551 Japan; 4 Institut de Sciences des Materiaux de Mulhouse (IS2M),CNRSUMR7361, Université de Haute-Alsace, 15, rue Jean Starcky, BP2488, Mulhouse Cedex 68057, France; 5 Laboratoire de Physique des Lasers, UMR 7538 du CNRS, Université Paris13-Sorbonne-Paris-Cité F-93430 Villetaneuse, France

**Keywords:** nano-scale, near-field etching, optically controlled magnetic-field interaction

## Abstract

Electric and magnetic fields play an important role in both chemical and physical reactions. However, since the coupling efficiency between magnetic fields and electrons is low in comparison with that between electric fields and electrons in the visible wavelength region, the magnetic field is negligible in photo-induced reactions. Here, we performed photo-etching of ZrO_2_ nano-stripe structures, and identified an etching-property polarisation dependence. Specifically, the etching rate and etched profiles depend on the structure width. To evaluate this polarisation-dependent etching, we performed numerical calculations using a finite-difference time-domain method. Remarkably, the numerical results revealed that the polarisation-dependent etching properties were determined by the magnetic field distributions, rather than the electric field distributions. As nano-scale structures induce a localised magnetic field, the discovery of this etching dependence on the magnetic field is expected to introduce a new perspective on advanced nano-scale structure fabrication.

## Introduction

Use of the near-field allows nano-scale imaging below the diffraction limit of light to be performed^1^. This technique has provided a means of visualising the point dipole in a single molecule^2^, centre-of-mass wavefunctions of an exciton in a quantum dot^3^, and molecular resolution imaging using pressure-assisted Raman imaging^4^.

Further, detailed study of electric fields in nano-scale materials has allowed the magnetic permeability in these substances to be tuned, while the recently developed nano-scale periodic-structure fabrication method^5^ has realised the predictions of Veselago^6^. In addition, the super-reflection property that originates from the negative refractive index has led to the realisation of super-resolution imaging and fabrication^7^. Many related applications have been developed, including optical cloaking^8^ and light harvesting devices^9^. However, although new phenomena have been observed in nano-scale materials as a result of adjustment of the magnetic permeability, the chemical and/or physical reactions within these substances are still determined by the electric field^7^. Further, the interaction of the magnetic field with the material excitation is considered to be negligible, because of the low coupling efficiency between magnetic fields and electrons.

In this study, nano-scale photo-etching of ZrO_2_ nano-stripe structures is performed, and the effects of the electric and magnetic fields on the etching properties are examined. Polarisation-dependent etching is observed, which is subsequently examined using a finite-difference time-domain (FDTD) approach.

## Materials and methods

### ZrO_2_ nano-stripe preparation

Polarisation-dependent nano-scale etching was performed on ZrO_2_ nano-stripe structures. The ZrO_2_ nano-stripe patterns were prepared using the deep-ultraviolet (DUV) interferometric lithography method reported in Ref. 10. This approach utilises a negative-tone resist based on photosensitive metal-oxo clusters, which are crosslinked and mineralised using DUV laser irradiation. Zirconium (IV) propoxide (Zr(OPr)_4_) (Sigma-Aldrich) was used as a metal alkoxide, and methacrylic acid (MMA) (Sigma-Aldrich) was used as a ligand to form a metal complex, with a molar ratio of Zr:MAA = 1:8. Water and isopropanol were added to adjust the physico-chemical properties of the resist. DUV irradiation led to the cross-linked structure to form ZrO_2_, after treatment of the non-exposed part with cyclohexanone^11^.

### Nano-scale etching

O_2_ in atmosphere was used as a source gas, and radical oxygen was used to etch the ZrO_2_^12^. To dissociate the O_2_, we used a continuous-wave (CW) He-Cd laser (λ = 325 nm; 3.81 eV; excitation power: 0.8 W cm^−2^). Thus, the incident photon energy was lower than the dissociation energy of the O_2_ (5.12 eV)^13^, and therefore, O_2_ dissociation occurred on the ZrO_2_ surface only. The absorption edge of a ZrO_2_ nano-stripe pattern is approximately 4.96 eV (250 nm)^11^; thus, we could exclude the effect of the carrier generation in the ZrO_2_ nano-stripe patterns. To evaluate the resultant changes in the three-dimensional ZrO_2_ structure, the surface structure was evaluated using an atomic force microscope (AFM) with a ‘Sampling Intelligent Scan’ mode (Nano Navi, Hitachi-Hitech-Science Corp., Japan).

## Results and discussion

**Figure 1:**
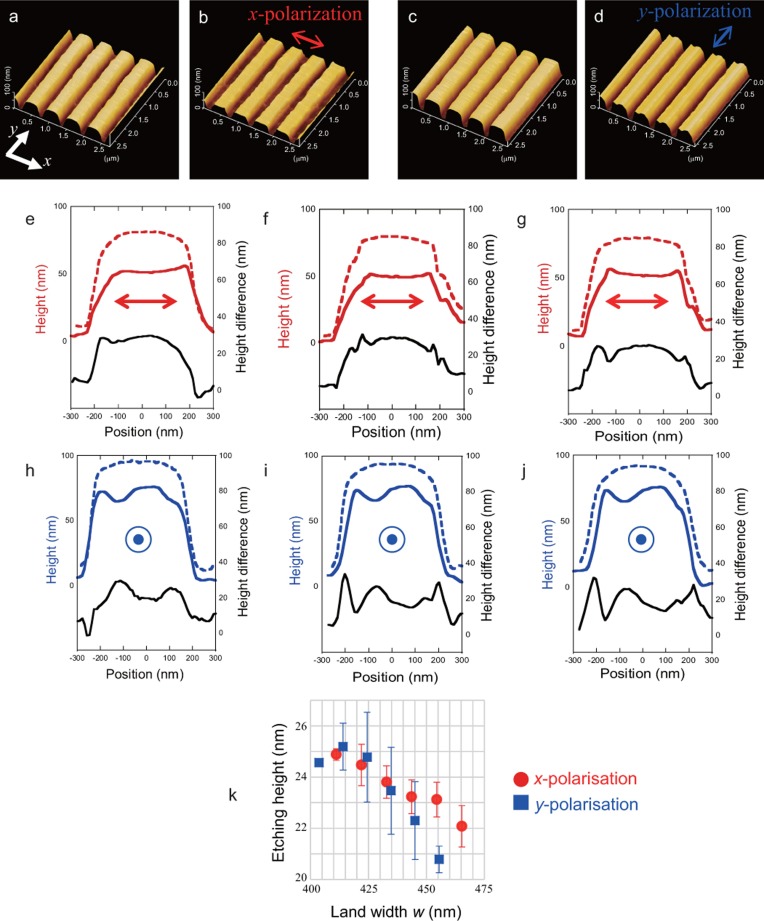
Polarisation-dependent etching. (**a**) and (**c**) are AFM images taken before etching. (**b**) and (**d**) are AFM images after 3 h. Perpendicular (*x*-polarised) and parallel (*y*-polarised) samples are shown in (**b**) and (**d**), respectively. Images (**a**) and (**b**), and (**c**) and (**d**), were obtained at the same respective positions. (**e–g**) Cross-sectional profiles of *x*-polarised samples. Red dashed line: pre-etched sample in (**a**), red solid line: etched sample in (**b**), black solid line: difference in height before and after etching. The pre-etched widths, *w*_b_, were (**e**) 411.17, (**f**) 432.81, and (**g**) 454.45 nm. (**h–j**) Cross-sectional profiles of *y*-polarised samples. Blue dashed line: pre-etched sample in (**c**), blue solid line: etched sample in (**d**), black solid line: difference in height before and after etching. *w*_b_ were (**h**) 414.06, (**i**) 424.41, and (**j**) 445.11 nm. (**k**) Etching height as a function of *w*.

Figure 1a and 1c show the AFM images obtained before etching. From the AFM images, the nano-stripe had a period of 598.4 ± 14.7 nm and a pre-etched width, *w*_b_, of 438.1 ± 13.0 nm. In addition, AFM images were taken at the same positions as those shown in Figure 1a and 1c, obtained after 180-min He-Cd laser irradiation with *x*- (perpendicular to the structure) and *y*- (parallel to the structure) polarisation, which are shown in Figure 1b and 1d, respectively. The incident light polarisation was controlled with a λ/2 waveplate. To evaluate the etching properties, we obtained the cross-sectional profiles in the *x*-direction in the AFM images (Figure 1a–1d). Figure 1e–1g and 1h–1j show typical cross-sectional profiles for *x*- and *y*-polarisation, respectively, taken at different *y*-positions. The dashed and solid curves correspond to images taken before and after etching, respectively, at the same *y*-position, while the black solid curve corresponds to the differences in the pre- and post-etching heights. From the differential height profiles, it is apparent that *x*-polarised etching resulted in uniform etching through the land area; however, the *y*-polarised etching resulted in non-uniform etching. To support this finding, we observed that the surface roughness, *R*_a_, at the land area was decreased from 1.14 to 0.60 nm in the *x*-polarisation case, while *R*_a_ was increased from 1.25 to 3.88 nm in the *y*-polarisation case. Additionally, we compared the etching height, that is, the average differential height through the land width, *w*, which was defined as the full width at 70% maximum of the pre-etched profile. Figure 1k shows the obtained etching height as a function of *w*, and indicates that the etching height decreased as *w* increased.

**Figure 2:**
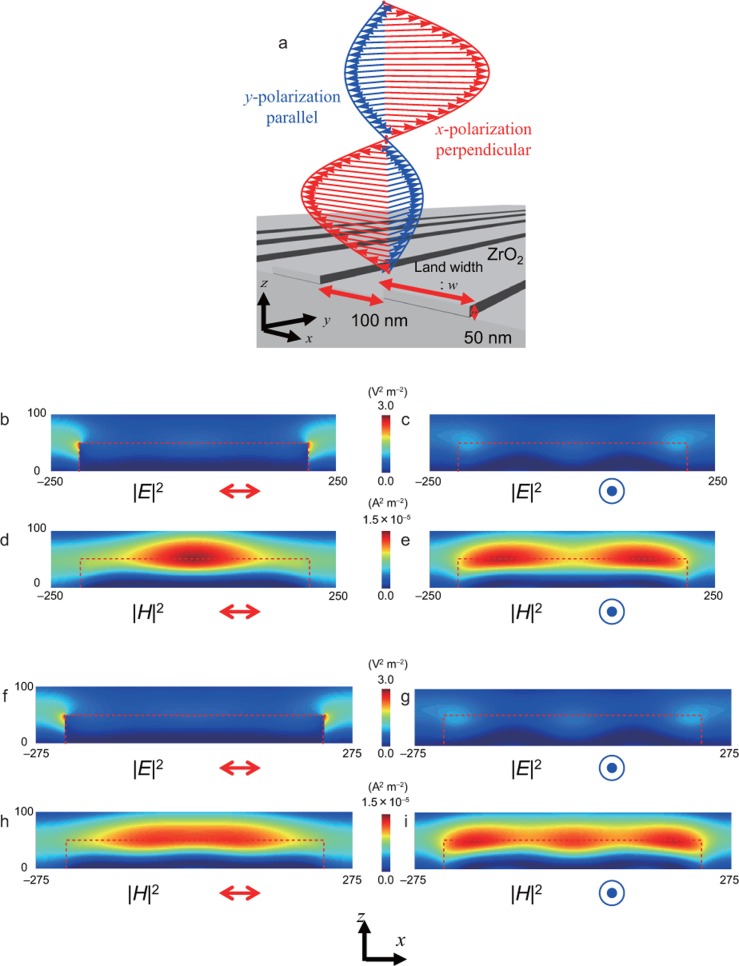
Size and polarisation dependence of electric- and magnetic-field intensity distributions. (**a**) Schematic of calculation model. (**b–e**) *w* = 400 nm. (**b**) |*E*|^2^ with *x*-polarisation. (**c**) |*E*|^2^ with *y*-polarisation. (**d**) |*H*|^2^ with *x*-polarisation. (**e**) |*H*|^2^ with *y*-polarisation. (**f–i**) *w* = 450 nm. (**f**) |*E*|^2^ with *x*-polarisation. (**g**) |*E*|^2^ with *y*-polarisation. (**h**) |*H*|^2^ with *x*-polarisation. (**i**) |*H*|^2^ with *y*-polarisation. The red dashed line corresponds to the ZrO_2_ land profiles.

To determine the etching-property dependence on the polarisation, we calculated the electric and magnetic field distributions using a FDTD method (Fujitsu Poynting for Optics). The cell size was 2 nm × 1 nm × 2 nm (corresponding to the *x*, *y* and *z* axes, respectively) and the model consisted of 600 × 1 × 2000 cells, with periodic boundary conditions imposed on the *x*- and *y*-axes and a perfectly matched layer (PML) absorbing boundary condition on the *z*-axis. A light source with a homogenous intensity distribution was set in the *xy*-plane 600 nm from the top surface of the ZrO_2_ structure (we used the ZrO_2_ refractive index of 1.8)^11^. The peak intensity of the incident electric-field was set to 1 V m^−1^. The ZrO_2_ structure model consisted of a periodic land- and space structure with 50-nm height, *w*-nm land width, and 100-nm spacing (Figure 2a).

Figure 2b–2i show the polarisation dependence of the electric and magnetic field distributions, with Figure 2b–2e and 2f–2i displaying the distributions for *w* = 400 and 450 nm, respectively. The electric field was localised at the corner of the land structure by the *x*-polarisation, independent of *w* (Figure 2b and 2f), while the magnetic field was focused in the vicinity of the land structure centre (Figure 2d and 2h). The distributions of both the electric and magnetic fields were dependent on the polarisation. Although the obtained ZrO_2_ nano-stripes were not precisely rectangular, as shown in the AFM images (Figure 1a and 1c), the calculated FDTD results for ZrO_2_ nano-stripes with tapered structures were almost identical to those for the rectangular structure model (see supplementary information). Thus, the use of a rectangular structure model to obtain the calculated results shown in Figure 2 is considered to be appropriate to explain the experimental results.

The photo-etching performed here used a CW He-Cd laser (3.81 eV), which has lower energy than the O_2_ photo-dissociation energy (5.12 eV). Furthermore, the incident laser power was too low to induce a non-linear energy up-conversion. However, many reports have demonstrated that the optical near-field induces energy up-conversion^14,15^, allowing the carrier excitation^16^ and the resultant chemical reaction^17^ to use less photon energy than molecule dissociation energy. Furthermore, direct observation of molecular dissociation using the near-field energy up-conversion has been reported^18^. Therefore, the nano-scale etching results shown in this manuscript should originate from the near-field-induced energy up-conversion.

**Figure 3:**
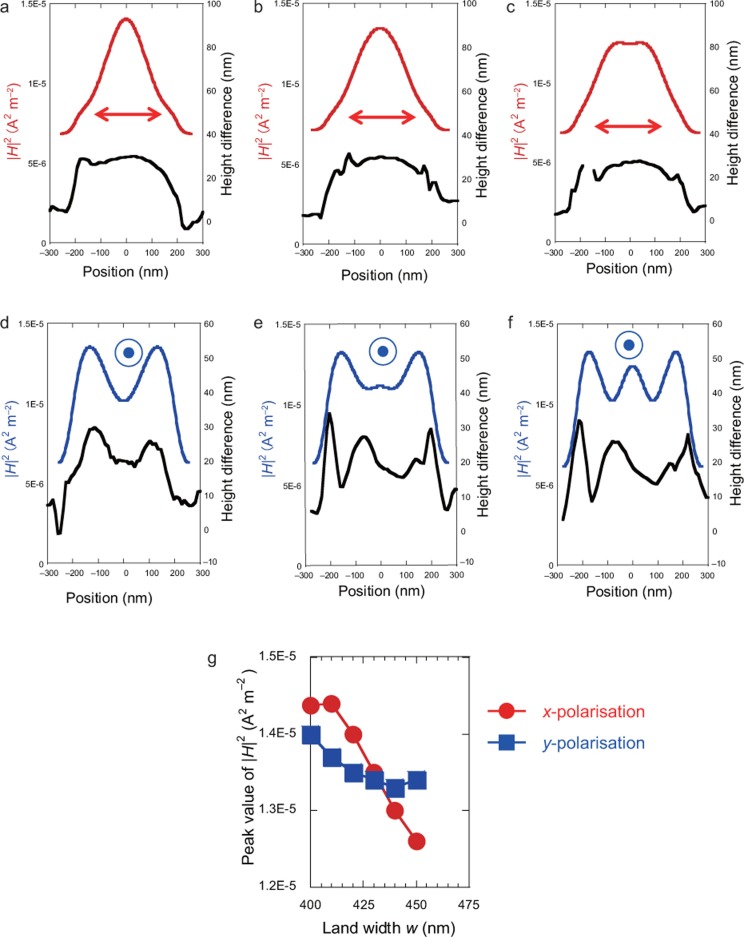
Comparison between pre- and post-etching heights. In (**a–f**), the red/blue and black solid lines correspond to the calculated |*H*|^2^ and the change in etching height, respectively. Further, the red and blue solid lines correspond to *x*- and *y*-polarisation, respectively. The calculated *w* and *w*_b_ with *x*-polarisation are (**a**) 410 and 411.17, (**b**) 430 and 432.81, and (**c**) 450 and 454.45 nm, respectively. The calculated *w* and the pre-etching widths, *w*_b_, with *y*-polarisation are (**d**) 410 and 414.06, (**e**) 430 and 424.41, and (**f**) 450 and 445.11 nm, respectively. (**g**) Peak values of calculated magnetic-field intensity as a function of *w*.

The electric field is localised at the corner of the land structure, which is due to the edge effect, and it may accelerate the etching rate. However, the calculated electric field distributions do not explain the polarisation dependence of the height difference. In contrast, the magnetic field is focused at the centre of the land structure. Figure 3a–3f show a comparison between the magnetic field distributions (the red and blue solid curves indicate *x*- and *y*- polarisation, respectively) and height differences (black solid curves). The results indicate that the height-difference profiles are in good qualitative agreement with the magnetic field distributions. That is, the etching profiles can be regarded as being governed by the magnetic field distributions, rather than the electric field distributions.

Additionally, as the nano-scale etching was realised by the localised magnetic field, the peak value of the magnetic field intensities was plotted as a function of the *w* (see Figure 3g), leading to decreased magnetic field intensity with increasing *w*. The dependence of the magnetic field intensity on *w* is similar to the experimental results for the etching height shown in Figure 1k. Taking the variations in the magnetic field distribution into consideration (see Figure 2d, 2e, 2h, and 2i), the increase in the etching rate as *w* decreases may contribute to higher magnetic-field concentration, with the land structure constructing the magnetic-field interference.

**Figure 4:**
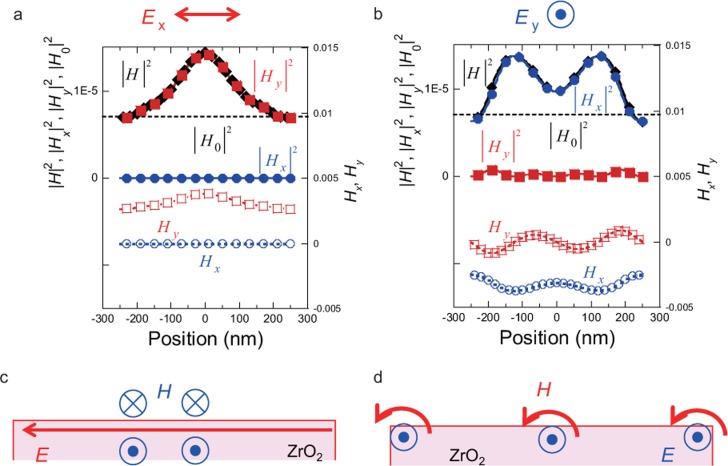
Magnetic field distributions depending on incident light polarisations. (**a**) *x*-polarisation, (**b**) *y*-polarisation. |*H*_0_| indicates the magnetic field intensity in the absence of nano-stripe structures. Schematic of magnetic-field generation for (**c**) *x*- and (**d**) *y*-polarisation. The calculated *w* is 400 nm.

To determine the magnetic field distributions shown in Figure 2, we observed the electric and magnetic field components. Figure 4a and 4b show the *x*- and *y*- components of the magnetic field for *x*- and *y*-polarisation, respectively, where the *z*-components were negligible for both types of polarisation. From these results, it is apparent that the magnetic field distributions due to *x*- and *y*-polarisation were determined by the *y*- (red solid squares in Figure 4a) and *x*- (blue solid circles in Figure 4b) components of the magnetic field, respectively. As is observed in Figure 2b (2f) and 2c (2g), the electric field is parallel to the incident light polarisation. The electric fields are generated as shown in Figure 4c and 4d, while the generated magnetic-field distributions (black solid diamonds in Figure 4a and 4b) are perpendicular to the incident light polarisation (Figure 4c and 4d). To evaluate the strength of the generated magnetic field, we compared the magnetic field intensity obtained with nano-stripe structures with that in free space, i.e., in the absence of nano-stripe structures (dashed black lines in Figure 4a and 4b). The peak magnetic field intensity generated using nano-stripe structures was twice that without them. As the polarisation dependence results for the nano-scale etching are qualitatively explained by the magnetic field distributions, rather than the electric field distributions, the nano-scale etching was governed by the localised magnetic field accompanying the energy up-conversion.

As described, the near-field etching originated from the radical oxygen species, which was dissociated from the O_2_ using the near-field energy up-conversion. The localisation of the optical near-field induces not only an electric dipole transition, but also an optically inactive transition including the magnetic dipole and magnetic quadrupole transitions, along with other higher-order transitions. In other words, the optical near-field allows activation of the optically inactive intermediate states^19,20^. As for the O_2_ potential energy curves, it has been reported that O_2_ has strong intermediate states such as 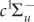 (transition energy from the ground state *E* = 4.05 eV) and 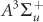 (*E* = 4.33 eV)^21^, which have transition energies from the ground state (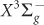) that are higher than the incident photon energy (*E*_1_ = 3.81 eV). Transitions from the ground states, that is, the magnetic quadrupole transitions (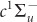
^22^ and 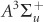
^21^), are all optically forbidden. Note that it is the second transition step from the intermediate to the excited state that is optically forbidden, i.e., the magnetic dipole transition (
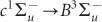
 and 
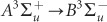
). The strength of the magnetic dipole transition depends on the intensity of the oscillating magnetic field. As the calculated magnetic field shown in Figure 2 is an oscillating field, the agreement of the height-difference profiles with the calculated magnetic field distributions shown in Figure 3 supports the finding that the localised magnetic field induces the nano-scale etching. This postulate is also supported by reports on the generation of the magnetic dipole moment by the near-field excitation, in which the magnetic dipole moment generation originates from the second harmonic electric field in the near-field^23^. Therefore, the localised magnetic field accompanying the energy up-conversion causes the chemical reaction via the optically inactive intermediate state.

## Conclusions

Through the nano-scale etching experiments described above, we discovered evidence of the strong magnetic-field interaction with the material excitation. Since conventional nano-scale fabrication using the near-field is designed considering the electric field only^7,24^, the incorporation of the magnetic field will provide an additional degree of freedom during the fabrication of advanced optical and/or electrical device structures on the nano-scale. Furthermore, the optically controlled magnetic-field interaction with material excitation on the nano-scale will allow new spin manipulation in space and time to be realised, in the absence of an external magnetic field^25^.
