# Supplementary material for: Optically controlled magnetic-field etching on the nano-scale
Source: Light Sci Appl. 2016 Mar 25;5(3):e16054–. doi: 10.1038/lsa.2016.54 (PMC6059895; doi:10.1038/lsa.2016.54)
Supplement: Supplementary information [file lsa201654x1.docx]

**Supplementary Information for**

**Optically controlled magnetic-field etching on the nano-scale**

Takashi Yatsui^1,*^, Toshiki Tsuboi^1^, Maiku Yamaguchi^1^, Katsuyuki Nobusada^2^, Satoshi Tojo^3^, Fabrice Stehlin^4^, Olivier Soppera^4^, and Daniel Bloch^5^

*1 School of Engineering, University of Tokyo, Bunkyo-ku, Tokyo, 113-8656 Japan*

*2 Department of Theoretical and Computational Molecular Science, Institute for Molecular Science, Myodaiji, Okazaki 444-8585 Japan*

*3 Faculty of Science and Engineering, Chuo University, Bunkyo-ku, Tokyo, 112-8551 Japan*

*4 Institut de Sciences des Materiaux de Mulhouse (IS2M), CNRS UMR 7361, Université de Haute-Alsace, 15, rue Jean Starcky, BP 2488, Mulhouse Cedex 68057, France*

*5 Laboratoire de Physique des Lasers, UMR 7538 du CNRS, Université Paris13-Sorbonne-Paris-Cité F-93430 Villetaneuse, France*

*Corresponding author: Yatsui T,
E-mail: yatsui@ee.t.u-tokyo.ac.jp

**1. FDTD analysis depending on the nano-stripe shape**

As the fabricated ZrO_2_ nano-striped were not perfectly rectangular, as shown in the AFM images in the main text (Fig. 1a and 1c), we performed calculations for tapered nano-stripe structures. To model the tapered structure, each corners of a 50-nm-high land was narrowed by 10 nm (Fig. S1a). Although the tapered structure resulted in rougher side walls in the FDTD model, comparison between the profiles for the electric (Fig. S1c and S1d) and magnetic fields (Fig. S1g and S1h) obtained for the rectangular and tapered structures indicates that significant differences do not exist. These results also indicate that the use of a rectangular structure model in the calculations is effective as regards explaining the findings.


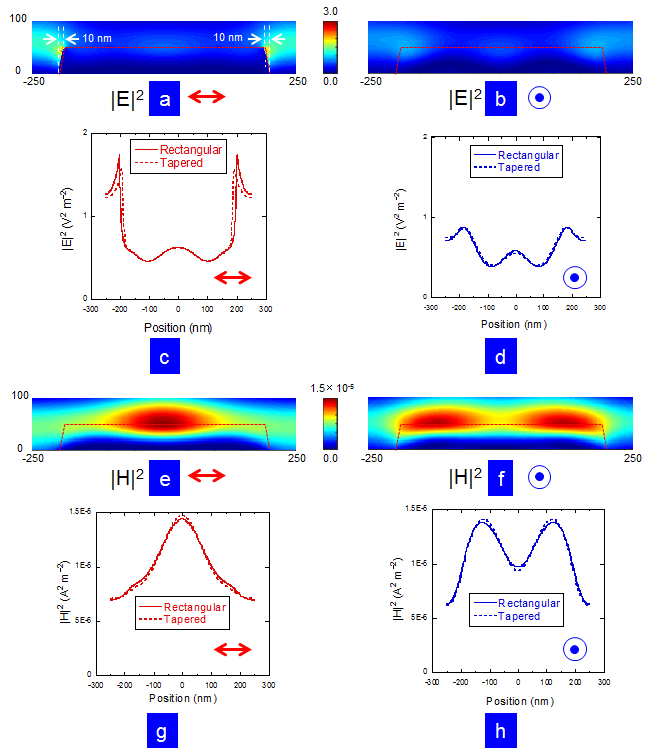


**Figure S1**  **Comparison of rectangular- and tapered-structure field distributions.** Tapered-structure electric field distributions for (a) *x*-polarisation and (b) *y*-polarisation. Comparison of electric-field profiles of rectangular (solid lines) and tapered (dashed lines) structures for (c) *x*-polarisation and (d) *y*-polarisation. Tapered-structure magnetic field distributions for (e) *x*-polarisation and (f) *y*-polarisation. Comparison of magnetic-field profiles of rectangular (solid lines) and tapered (dashed lines) structures with (g) *x*-polarisation and (h) *y*-polarization.
